# Septic arthritis of the native hip joint: a multi-pattern, multi-outcome disease

**DOI:** 10.1007/s00590-023-03477-2

**Published:** 2023-01-29

**Authors:** Byungseob Kim, Baptiste Boukebous, Douglas White, Joseph F. Baker

**Affiliations:** 1 Faculty of Medical and Health Science, University of Auckland, Grafton, Auckland, New Zealand; 2 Department of Orthopaedic Surgery, Waikato Hospital, Hamilton, New Zealand; 3 ECAMO Team, CRESS, UMR1153, INSERM, Paris, France; 4 Department of Rheumatology, Waikato Hospital, Hamilton, New Zealand; 5 Department of Medicine, University of Auckland, Auckland, New Zealand; 6 Department of Surgery, University of Auckland, Auckland, New Zealand

**Keywords:** Septic arthritis, Arthrotomy, Native hip joint, Systemic inflammatory diseases, Mortality, Complication

## Abstract

**Purpose:**

Septic arthritis of the native hip joint (SANH) is an uncommon surgical and medical emergency with few reports. The aim of this study was to determine predictors of return to theatre (RTT), complications and mortality.

**Methods:**

Patients with SANH were identified from January 2009 to June 2022; 50 patients and three subgroups were identified: *Pyogenic* (surgical washout without systemic inflammatory disease), *Systemic* (surgical washout with SIDs) and patients managed non-surgically*.* Patterns of these groups were assessed with a principal component analysis. The cumulative incidences for death, any complication and RTT for repeat washout were calculated. The predictive variables associated with outcomes were selected with univariable models and then incorporated in multivariable CoxPH regressions.

**Results:**

The 1-year cumulative incidence was 14% for mortality and 48.5% for any complication. Amongst patients managed surgically, 1-year risk of RTT was 46% in *Pyogenic* subgroup and 21% in *Systemic* subgroup. *Systemic* subgroup had lower complications and RTT and higher rate of sterile aspirate, compared to *Pyogenic.* Charlson comorbidity index (CCI) (HR = 1.41, *P* value = 0.03), preoperative albumin (HR = 0.81, *P* value = 0.009) and preoperative haemoglobin (HR = 0.95, *P* value = 0.02) were significantly associated with 1-year mortality. Time between symptom onset and admission > 7 days (HR = 3.15, *P* value = 0.042), preoperative Hb (HR = 1.05, *P* value = 0.016), socioeconomic deprivation (HR = 1.18, *P* value = 0.04) and *Systemic* subgroup (HR = 0.25, *P* value = 0.04) were significantly associated with RTT.

**Conclusion:**

Mortality was well predicted by the usual parameters including CCI, albumin, but also low haemoglobin. Patients presenting in a delayed fashion were more likely to have multiple lavages.

## Introduction

SA is a potentially life-threatening condition with reported mortality of 11–19% [1, 2]. It is a surgical emergency that requires prompt assessment, diagnosis and treatment.

The current largest study of native joint SA in adults reported incidence of 21/100,000 with pronounced differences between ethnicity [3].

There is a relative paucity of literature focussing on SA of the native hip (SANH) in adults as studies often include periprosthetic joint infections [1, 2, 4] and paediatric population [1, 4, 5]. Cohorts involving SA of the native hips in adults usually have a limited number of participants, often less than 30 [6–8].

Our experience suggests that patients with SANH have different patterns, associated with different outcomes in terms of repeat washout, complications and mortality rate. Particularly, a number of patient with SANH have a negative culture despite the presence of macroscopic pus and have an associated diagnosis of systemic inflammatory disease (SID). These different patterns need to be further described.

Given the current limited findings, we performed a retrospective review of native hip SA in adults. Our aim was to identify predictors for complications, return to theatre (RTT) and mortality.

## Materials and methods

This retrospective cohort study was conducted in a large tertiary teaching hospital covering more than 21,000 km^2^. No ethics approval was deemed necessary as no patient contact initiated.

## Data sources

**Fig. 1:**
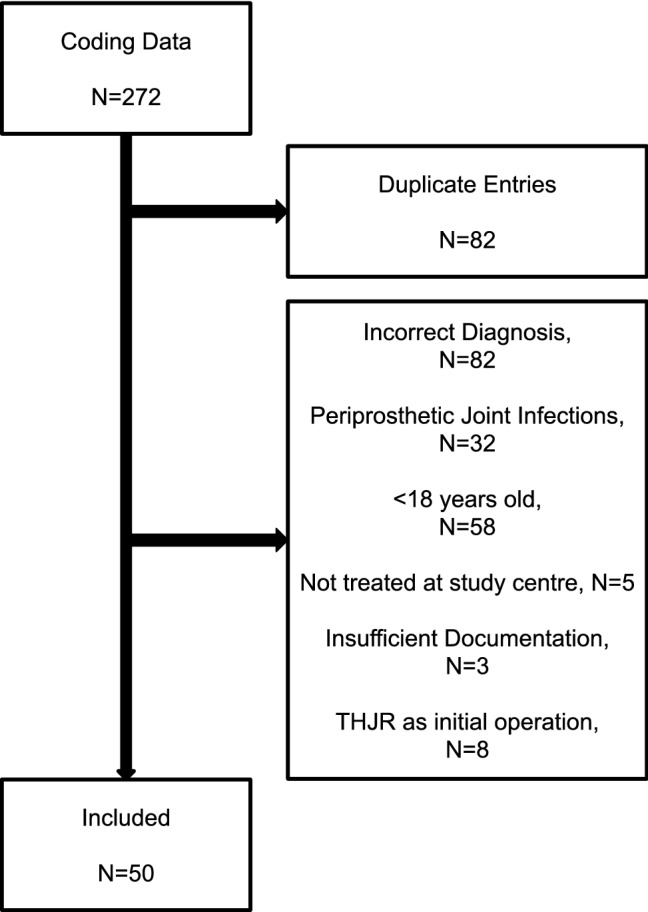
Flow chart for inclusion criteria

Patients were identified using the ICD-10 code from January 2009 to June 2022. Cases were classified using the Newman’s criteria for the diagnosis of SA. [5]. Exclusion criteria are shown in Fig. 1.

## Data collection

### Demographics collected include

Age, gender, ethnicity, smoking status and socioeconomic deprivation status. Socioeconomic deprivation status was collected using the New Zealand Deprivation Index 2018 (NZDep18), ranging from 1 to 10, least deprived to most deprived, respectively.

### Presenting data collected include

Presenting complaints, number of days between symptom onset and operation/presentation to hospital (for patients treated nonoperatively), length of stay (LOS) and infections at other sites.

### Comorbidities collected include

smoking status, osteoarthritis, intravenous drug use, immunosuppressant use and conditions involved in the Charlson comorbidity index (CCI). Systemic inflammatory disease (SID), diagnosed by a relevant medical specialist, was also reported.

### Investigation results include

Haemoglobin (Hb), albumin (Alb), lymphocyte count, platelet lymphocyte ratio (PLR), white cell count (WCC), C-reactive protein (CRP). Blood and urine culture results and joint aspiration results were also collected.

Outcomes of interest were any complication, return to theatre (RTT) and mortality. RTT was defined as need for repeat washout of the hip—this being determined by the responsible consultant surgeon for the patient. The complications were classified using the Clavien–Dindo classification system. The date of last follow-up (LFU) was the date of the last clinical assessment, for any reason.

## Bias

The main source of bias addressed was the missing values, and the count of those is reported in Table 1. Multiple imputation using the Multivariate Imputation by Chained Equation (MICE) approach was performed to reconstruct the missing values (function *mice()*, *R* package *mice* 2.46). A total of six datasets were analysed, one being the “native” dataset with the missing values (for complete case analysis) and five imputed datasets. Sensitivity analysis was conducted for every model, to assess the effect of the unobserved data. The models were run with the six datasets, and the outcomes were considered robust if the coefficients and *p*-values remained similar under the six alternatives scenarios.

**Table 1:** Descriptive data (*N* = 50). Data are *N* (%) unless otherwise stated

Age, years, median (Q1–Q3)	60 (38–70)
CCI, median (Q1–Q3)	3 (1–5)
NZDep18, median (Q1–Q3)	8 (5–10)
Preop Hb, g/L, median (Q1–Q3)	119 (103–130)
Preop Alb, g/L median (Q1–Q3)	32 (24–35)
Preop WCC, × 10^9^/L, median (Q1–Q3)	11 (8–13)
Preop CRP, mg/L, median (Q1–Q3)	132 (78–280)
CRP day 3, mg/L, median (Q1–Q3)	110 (62–167)
CRP day 5, mg/L, median (Q1–Q3)	51 (14–99)
CRP trend	
Fall	40 (85)
Plateau	6 (13)
Rise	1 (2)
Preop lymphocytes, × 10^9^/L, median (Q1–Q3)	1 (1–2)
Preop PLR, median (Q1–Q3)	222 (148–426)
Aspirate WCC, × 10^6^/L, median (Q1–Q3)	36,600 (10,800–87,250)
Days between symptom onset and presentation, median (Q1–Q3)	4 (2–7)
Gender	
Female	19 (38)
Male	31 (62)
Smoking status	
Non-smoker	16 (50)
Current smoker	9 (28)
Ex-smoker	7 (22)
Ethnicity	
NZ Maori	20 (40)
NZ European	23 (46)
Other	7 (14)
Immunosuppression	
No	41 (82)
Yes	9 (18)
Diabetes mellitus	
No	45 (90)
Yes	5 (10)
Infections at other site	
No	28 (56)
Yes	22 (44)
Newman’s criteria	
A	32 (64)
B	4 (8)
C	14 (28)
Aspirate microbiology	
No growth	10 (20)
No growth with preop antibiotics	6 (12)
Other	6 (12)
Staphylococcus	21 (43)
Streptococcus	6 (12)
Systemic inflammatory disease	
No	35 (70)
Yes	15 (30)

The second source of bias was death, acting as a competing risk, while estimating the risks of RTT and complications. A thorough assessment of mortality was performed; the software providing the electronic records is interconnected with the other healthcare providers in the community so it allows an accurate and updated assessment of mortality. The last assessment of mortality was done in July 2022.

## Statistics

Analyses were carried out on R software (R 3.6.0, R Foundation for Statistical Computing).

A Principal Component Analysis (PCA) was carried out to provide a visual representation of the three subgroup’s patterns. The correlations were confirmed with a correlation test.

The primary outcome was the assessment of the complications, RTT and death in terms of incidence and risk factors. The cumulative incidence of each of these events was estimated with the cumulative incidence estimator, up to 1-year follow-up. Cumulative incidences were compared in the three subgroups, and Gray’s test was performed in case of competing risk.

The following variables were tested in univariable models: age, CCI, blood sample figures, aspiration WCC (continuous), gender, subgroups, smoking status, blood transfusion, Newman’s classification, NZDep18, SIDs, ethnicity, aspiration culture (dichotomous). Those reaching some degree of significance with a log rank test (*P* < 0.2) were entered in a CoxPH multivariable regression model, providing hazard ratios (HR). The primary alpha risk was set at 0.05.

## Results

### Participants and descriptive data

A total of 50 patients and three subgroups were identified. The first subgroup, ‘*Pyogenic*’*,* corresponded to 29 patients with a surgical washout and no history of SID. The second subgroup, ‘*Systemic*’*,* comprised 15 patients with or without surgical washout and with diagnosis of SID. The third subgroup, ‘*Non-Op’,* corresponded to 6 patients managed nonoperatively; 3 patients were unfit for surgery due to comorbidities, 2 patients clinically improved by the time surgical team reviewed, and 1 patient was planned for surgery, however, deteriorated due to pneumonia and was deemed unfit for anaesthesia.

Demographics are described in Table 1. The mean age was 56.1 yo (SD: 20), 19 females and 31 males. The mean follow-up was 51 months (SD: 46, median 39, Q1: 14, Q3: 79). By July 2022, 22 patients deceased.

Fifteen patients had SID; amongst them, fourteen patients were treated surgically. Eight patients had seropositive rheumatoid arthritis, two patients had seronegative polyarthritis, one patient had ankylosing spondylitis, and one patient had positive antinuclear antibodies (not further specified). Two other patients had ulcerative colitis (one with a colectomy), and one more had chronic diarrhoea, duodenitis, gastritis and B12 deficiency. For four patients, SANH was the first presentation of SID.

**Fig. 2:**
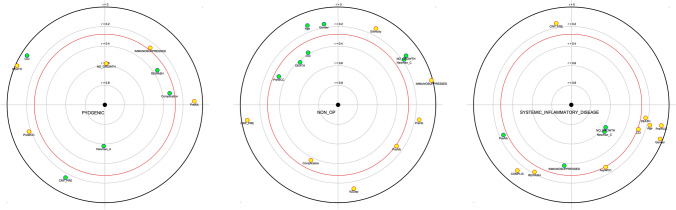
Demographic characteristics of subgroups *R* plot. The yellow dots correspond to negative correlation and the green dots positive correlation. The coefficient of correlation is given by the concentric circles, and the closer the dot is from the centre, the higher the association is. The red circle represents significance (*P* value < 0.05). When several explicative variables are correlated between each other, they are clustered together. When explicative variables are negatively associated, they are diametrically opposed

The PCA showed the three subgroups had distinguished demographic characteristics (Fig. 2). The *Pyogenic* subgroup was significantly associated with Newman’s A (*r *= 0.57, *P* value < 0.001), RTT (*r* = 0.35, *P* value = 0.01) and complications (*r* = 0.32, *P* value = 0.02). The *Systemic* subgroup was significantly associated with Newman’s C and sterile cultures (*r* = 0.58, *P* value < 0.001). On admission, four patients of the *Systemic* subgroup had macroscopic frank pus, and five had turbid/straw-coloured synovial fluid. Three patients of the *Systemic* subgroup had RTT, but no collection was found. A total of 16/50 patients had sterile culture; amongst them 11/16 were in the group *Systemic* (*P* value = 0.0002). Patients of subgroup *Non-Op* were significantly associated with high CCI (*r* = 0.38, *P* value = 0.005), mortality (*r* = 0.42, *P* value = 0.002) and higher WCC in aspirate (*r* = 0.33, *P* value = 0.02).

## Main results

**Fig. 3:**
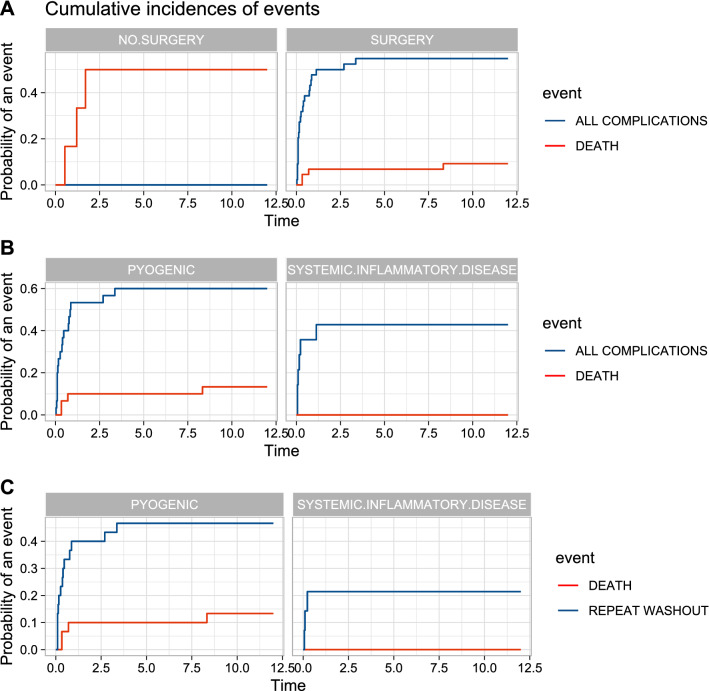
1-year cumulative incidences of events. Time is in months

The 1-year cumulative incidence was 14% (95% CI 4.2–23.9%) for mortality, 48.5% (95% CI 34–-62%) for any complication. The HR for mortality was 3.38 (95% CI 1.2–8.9) for the *Non-Op* subgroup, Fig. 3a. Amongst the 44 patients with surgical treatment, the overall 1-year cumulative incidence for any complication was 55% (95% CI: 39%-70%), 60% (95% CI: 42%-78%) in *Pyogenic*, 42% (95% CI 15–70%) in *Systemic*, but Gray’s test was not significant (*P* value = 0.16), Fig. 3b. Still amongst patients managed surgically, 1-year risk of RTT was 39% (95% CI 24–53%), 46% (95% CI 28–65%) in *Pyogenic* and 21% (95% CI 0–43%) in the *Systemic*, but Gray’s test failed to reach significance (*P* value = 0.17), Fig. 3c.

The following variables had some association with risk of any complication and were selected for a multivariable regression: NZDep18, days between symptom onset and admission, and subgroup *Non-Op*. However, none of these variables were significantly associated with complications in the multivariable model. The sensitivity analysis did not change the results for the complications.

The following variables had some association with the risk of death at 1-year: CCI score (including age), immunosuppression, multi-location of infection, preoperative Hb and Alb, and days between symptom onset and admission. In multivariable models, the CCI score (HR = 1.41, *P* value = 0.03), preoperative Alb (HR = 0.81, *P* value = 0.009) and preoperative Hb (HR = 0.95, *P* value = 0.02) remained significantly associated with 1-year mortality. These results were robust with the sensitivity analysis.

The following variables had some association with the risk of RTT for repeat washout (Table 2), amongst patients with surgical management: NZDep18, preoperative Hb, days between symptom onset and admission, and subgroup *Systemic*. In multivariable models, time between symptom onset and admission above 7 days (HR = 3.15, *P* value = 0.042), preoperative Hb (HR = 1.05, *P* value = 0.016), NZDep18 (HR = 1.18, *P* value = 0.04), and *Systemic* subgroup (HR = 0.25, *P* value = 0.04) were significantly associated with RTT. These results were robust with the sensitivity analysis. The mean preoperative Hb for patients who were treated surgically and died rapidly without having sustained a repeat washout was 97 g/L. The mean preoperative Hb of patients treated surgically and who did not die within 1-year postoperatively was 120 g/L.

**Table 2:** Risk of RTT for repeat washout. 95% confidence interval in brackets

	Univariable analysis		Multivariable analysis*	
Variables	Hazard ratio	*P* values	Hazard ratio	*P* values
Age	0.99 (0.97–1.02)	0.48	–	–
CCI	0.97 (0.81–1.17)	0.76	–	–
NZDep18	1.14 (0.94–1.39)	0.17	1.18 (0.96–1.46)	0.04
Preop Hb	1.02 (0.99–1.05)	0.14	1.05 (1.01–1.08)	0.016
Preop Alb	1.04 (0.96–1.12)	0.31	–	–
Preop WCC	0.98 (0.92–1.04)	0.48	–	–
Preop CRP	1 (1–1)	0.78	–	–
TimePre**	1 (0.99–1.02)	0.4	1.01 (1–1.02)	0.074
TimePre > 7 days				
No	REF	REF	–	–
Yes	1.90 (0.7–5.18)	0.19	3.15 (1.04–9.73)	0.042
Preop Lymphocytes	0.93 (0.69–1.25)	0.47	–	–
Preop PLR	1 (1–1)	0.47	–	–
Aspirate WCC	1 (1–1)	0.3	–	–
Gender				
Female	REF	REF	–	–
Male	1.35 (0.5–3.62)	0.55	–	–
Smoker	–	–	–	–
Non-smoker	REF	REF	–	–
Current smoker	0.61 (0.2–2.29)	0.46	–	–
Ex-smoker	0.94 (0.2–3.54)	0.93	–	–
Immunosuppression***				
No	REF	REF	–	–
Yes	2.27 (0.8–6.42)	0.12	–	–
Diabetes mellitus				
No	REF	REF	–	–
Yes	1.92 (0.5–6.6)	0.30	–	–
Infections at other site				
No	REF	REF	–	–
Yes	1.05 (0.41–2.68)	0.91	–	–
Newman’s criteria	–	–	–	–
A	REF	REF	–	–
B	0.52 (0.1–4)	0.53	–	–
C	0.75 (0.2–2.3)	0.61	–	–
CRP trend				
Fall	REF	REF	–	–
Plateau	0.47 (0.1–3.59)	0.47	–	–
Rise	3.05 (0.4–23.55)	0.29	–	–
Aspirate microbiology				
No growth	0.53 (0.1–5.15)	0.59	–	–
No growth with preop antibiotics	0.53 (0.1–5.15)	0.59	–	–
Other	1.47 (0.3–7.31)	0.64	–	–
Staphylococcus	1.27 (0.3–4.78)	0.73	–	–
Streptococcus	1.03 (0.2–6.17)	0.97	–	–
Systemic inflammatory disease				
No	REF	REF	REF	REF
Yes	0.42 (0.1–1.47)	0.18	0.25 (0.06–0.96)	0.04

*Results were pulled running the models in the six different datasets, amongst which five scenarios to explore the sensitivity to the missing values

**Time between symptom onset and admission. This was tested as a continuous variable, and a binary variable (> or < 7 days), in two different models

***Immunosuppression was not entered in the models to avoid collinearity as it is highly associated with SID

## Discussion

In this retrospective analysis of 50 cases, strong predictors for RTT were identified: time between symptom onset and presentation, socioeconomic deprivation and absence of SID. Preoperative Hb was also a statistically significant result; however, it is unlikely to be true.

Delayed presentation of longer than 7 days and socioeconomic deprivation were shown to be a strong predictor of RTT. This is consistent with what is already known for SA. It has been previously reported consistently that delay in management results in poorer outcomes [9–11]. Our study confirms current findings and stresses the importance of prompt management in a timely manner.

A group of patients with any SID had reduced risk of complications and RTT, and they accounted for 11 out of 16 sterile cultures. Rheumatoid arthritis is a risk factor for SA [3, 12], and it was previously reported to have poorer outcome of the affected joint [13] and increased recurrence of SA [14]. However, our results show that patients with SID had reduced risk of RTT compared to patients without SIDs. Kennedy et al. reported that there was no difference in the distribution of Newman’s criteria and proportion of organisms in SA patients with rheumatoid arthritis [15] which is different to our findings. We suggest testing for and identifying patients with SID as they can mimic SA. Rheumatic conditions such as psoriatic arthritis and crystal arthritis can also have raised aspirate WCC and fever [16–18]. Treatment for these conditions may include steroids or more powerful immunosuppression which can delay the presentation and create diagnostic confusion by reducing inflammatory markers or WCC. We also must question the need for washouts for patients with SID and sterile culture. As these patient groups tend to have better outcomes, urgent aspiration and antibiotics may be enough as initial management.

Preoperative Hb was found to be a statistically significant result for rate of RTT. The higher preoperative Hb the patient had, the more likely the patient to RTT for repeat washout, which was an unexpected result. Patients who died soon after initial washout had a lower mean Hb of 97 g/L compared to 120 g/L in patients who did not die within 1-year postoperatively. Low Hb was a strong predictor for mortality. Patients with low Hb passed away soon after initial washout; subsequently, they could not sustain any RTT. On the other hand, patients who had higher Hb likely survived longer and had time to sustain RTT for ongoing infection. All in all, high Hb as a risk factor of RTT is probably not relevant and the result of competition. The genuine result to consider is low Hb as a risk factor for death. This highlights the importance of competing risk analysis, especially in case of high mortality rate.

Another explanation for the high Hb is it may indicate dehydration as Hb is measured as concentration. Along with low Alb, this may indicate sepsis [19, 20]. Also, as sepsis causes destruction of erythrocytes, haemoglobin is released into the bloodstream which gets oxidised to free haem. Free haem increases cell death and exacerbates inflammation [21]. Due to this, patients may have clinically deteriorated, indicating RTT for repeat washout.

Patients were also checked if they received recent blood product transfusions as they can cause an increase in Hb and increase risk of infection [22]. However, there was no statistical correlation upon analysis. There is also a possibility of confounding factors playing a role that were not measured in this study.

Diabetes is a recognised risk factor for SA [3], and different immunosuppressants have different infection risks [23]. Formal analysis of these variables would be valuable. However, due to limited number of patients and lack of power, statistically significant results were not obtained.

40% of our patients were NZ Maori. Although NZ Maori were reported to have twice the incidence rate compared to NZ European [3], NZ Maori may have been over-represented in our study as 23% of our institution’s population is NZ Maori (compared to the national average of 16%).

Our study had comprehensive follow-up, mean follow-up of 51 months. We did not lose any patients and had a comprehensive assessment of mortality. Although 22 patients were deceased by July 2022, this was not all due to SANH. Hence, 1-year mortality was used for formal analysis. This makes our study results robust and unique to other studies.

## Limitations

The main limitation is the lack of power. The Gray’s test did not reach significance although there were strong differences between the subgroups, probably because of lack of events. Similarly, some variables were very close to the significance in the multivariable models. The lack of patients limited the number of explicative variables in the multivariable models, and the interactions between variables were difficult to address. Furthermore, some explicative variables had few missing values. Without imputation, the number of observations in the complete case analyses plummeted even more. Nevertheless, the results obtained after imputation were tested in sensitivity analyses, and remained robust, with similar coefficient and close *p* values.

## Conclusion

Mortality was well predicted by the usual parameters including CCI, Alb, but also low Hb. Delayed presentation to hospital increased the risk of needing multiple trips to the operating room. Patients with systemic inflammatory diseases were also found to be less likely to return to theatre and had reduced complications. Some patients, presenting with a septic arthritis, were actually presenting with a first episode of SID. Hence, we suggest systematic screening for SID, for patients with no known history.
